# Physical Confirmation and Comparative Genomics of the Rat *Mammary carcinoma susceptibility 3* Quantitative Trait Locus

**DOI:** 10.1534/g3.117.039388

**Published:** 2017-04-05

**Authors:** Saasha Le, Zachary C. Martin, David J. Samuelson

**Affiliations:** * Department of Biochemistry and Molecular Genetics, University of Louisville School of Medicine, Kentucky 40292; † James Graham Brown Cancer Center, University of Louisville School of Medicine, Kentucky 40202; ‡ Center for Genetics and Molecular Medicine, University of Louisville School of Medicine, Kentucky 40202

**Keywords:** rat QTL, breast cancer susceptibility, mammary carcinogenesis

## Abstract

Human breast and rat mammary cancer susceptibility are complex phenotypes where complete sets of risk associated loci remain to be identified for both species. We tested multiple congenic rat strains to physically confirm and positionally map rat *Mammary carcinoma susceptibility 3* (*Mcs3*)—a mammary cancer resistance allele previously predicted at *Rattus norvegicus* chromosome 1 (*RNO1*). The mammary cancer susceptible Wistar Furth (WF) strain was the recipient, and the mammary cancer resistant Copenhagen (Cop) strain was the *RNO1*-segment donor for congenics. Inbred WF females averaged 6.3 carcinogen-induced mammary carcinomas per rat. Two WF.Cop congenic strains averaged 2.8 and 3.4 mammary carcinomas per rat, which confirmed *Mcs3* as an independently acting allele. Two other WF.Cop congenic strains averaged 6.6 and 8.1 mammary carcinomas per rat, and, thus, did not contain *Mcs3*. Rat *Mcs3* was delimited to 27.8 Mb of *RNO1* from *rs8149408* to *rs105131702* (*RNO1*:143700228-171517317 of RGSC 6.0/rn6). Human genetic variants with *p* values for association to breast cancer risk below 10^−7^ had not been reported for *Mcs3* orthologous loci; however, human variants located in *Mcs3*-orthologous regions with potential association to risk (10^−7^ < *p* < 10^−3^) were listed in some population-based studies. Further, rat *Mcs3* contains sequence orthologous to human *11q13/14*—a region frequently amplified in female breast cancer. We conclude that *Mcs3* is an independently acting mammary carcinoma resistance allele. Human population-based, genome-targeted association studies interrogating *Mcs3* orthologous loci may yield novel breast cancer risk associated variants and genes.

Excluding skin cancer, breast cancer is the most commonly diagnosed cancer among women in the United States, accounting for 29% of cancers diagnosed, and 14% of cancer-related deaths in females (Siegel *et al.* 2016). The current population-based lifetime-risk of a woman being diagnosed with breast cancer is 12–13% (Siegel *et al.* 2016). Breast cancer susceptibility is a complex trait controlled by genetic, epigenetic, and environmental factors. The genetic component of breast cancer susceptibility is comprised of a spectrum of high, moderate, and low penetrance risk associated mutations and variants (Pharoah *et al.* 2002). Genetic linkage analyses and genome-wide association studies (GWAS) have enabled the identification of several risk alleles, but a majority of susceptibility loci remain unidentified (Smith *et al.* 2006; Ioannidis *et al.* 2010; Mavaddat *et al.* 2010; Economopoulou *et al.* 2015). Furthermore, GWAS have yielded many potentially associated variants. These are polymorphisms with *p* values for association above a stringent genome-wide significance threshold level of 1 × 10^−7^, which is commonly used to avoid false-positive associations (Thomas *et al.* 2005). Comparative genomics approaches that integrate relevant QTL of experimental organisms and human loci, containing potentially associated variants, may be useful to identify additional risk associated alleles. To serve this approach, mammary cancer susceptibility QTL identified in experimental organisms must be mapped to conserved syntenic regions to effectively guide human genome-targeted association studies (Lesueur *et al.* 2005; Samuelson *et al.* 2007).

Laboratory rats (*Rattus norvegicus*) provide a good experimental model of female breast cancer, as rat mammary carcinomas are similar to human breast carcinomas with respect to histopathology and hormone responsiveness (Gould 1995). Evidence suggests that both rat mammary and human breast carcinomas originate from mammary ductal cells (Russo *et al.* 1983). Inbred rat strains that differ in susceptibility to 7,12-dimethylbenz[a]anthracene (DMBA)-, N-methyl-N-nitrosourea (NMU)-, or estrogen-induced mammary carcinogenesis have been used to predict locations of *Mammary carcinoma susceptibility* (*Mcs*) and *Estrogen-induced mammary cancer* (*Emca*) loci (Hsu *et al.* 1994; Shepel *et al.* 1998; Lan *et al.* 2001; Gould *et al.* 2004; Quan *et al.* 2006; Schaffer *et al.* 2006; Ren *et al.* 2013). Linkage analyses using crosses between the DMBA-induced mammary carcinoma susceptible Wistar-Furth (WF) and resistant Copenhagen (Cop) strains resulted in four predicted *Mcs* QTL named *Mcs1*, *Mcs2*, *Mcs3*, and *Mcs4* (Hsu *et al.* 1994; Shepel *et al.* 1998). Resistance-associated QTL, *Mcs1* and *Mcs2*, have been physically confirmed (Haag *et al.* 2003; Sanders *et al.* 2011). In this article, we report our congenic strain results that physically confirm rat *Mcs3*, and delimit this Cop resistance QTL to a 27.8 Mb segment of rat chromosome 1 (*RNO1*).

## Materials and Methods

### Congenic breeding and genotyping

Congenic lines were made by adapting previously described methods to *RNO1* (Samuelson *et al.* 2003). Briefly, rats with selected Cop *RNO1* segments from the predicted *Mcs3* QTL were introgressed into a WF/NHsd genetic background by successive backcrossing to the WF strain. Congenic rat lines were maintained in an Association for the Assessment and Accreditation of Laboratory Animal Care (AAALAC)-approved facility on a 12-hr light/dark cycle, and provided LabDiet 5001 Rodent Diet (PMI Nutrition International) and water *ad libitum*. All animal protocols were approved by the University of Louisville Animal Care and Use Committee. Sequence information and locations of genetic markers defining the ends of COP alleles in each congenic line A, D, E, and G are available at the UCSC Genome Browser (www.genome.ucsc.edu), the Rat Genome Database (http://rgd.mcw.edu/), or Supplemental Material, Table S1. Animals were genotyped as described in Samuelson *et al.* (2003). Briefly, tail clips were used for DNA extraction and genotyping by either gel electrophoresis or Sanger sequencing of PCR products. Informative genetic markers at respective congenic ends, and at 10 Mb intervals between ends, were used to determine genotypes. For microsatellite markers, each fast-PCR underwent denaturation of 95° for 10 sec, followed by 40 cycles of 94° for 0 sec and 63° for 8 sec, and an extension at 72° for 30 sec on an Applied Biosystems Veriti Fast Thermal Cycler. **A**mplified genomic DNA was run on a 3% high-resolution agarose gel. After electrophoresis, gels were stained in SYBR Gold and scanned using a Typhoon imager. Visible gel-bands of the appropriate size were analyzed along with DNA from homozygous Cop and WF, and (WF × Cop)F_1_ (heterozygous) rats. In regions where informative microsatellite markers were limiting, Primer3 was used to design primers to PCR amplify genomic regions containing potentially WF/Cop informative single nucleotide variants (SNVs), identified using the SNPlotyper function available at the Rat Genome Database. Genomic DNA was extracted from spleen tissue, and PCR amplified. These reactions were cleaned using Promega Wizard SV Gel and PCR clean-up system. Samples were sequenced by the University of Louisville, Center for Genetics and Molecular Medicine, sequencing core using an ABI PRISM 7700 Sequence Detection System. Primer sequences that define the ends of *Mcs3* congenic lines A and E can be found in Table S2.

### Phenotyping

Female WF.Cop congenic and WF/NHSd (Envigo) rats were gavaged with a single oral dose of DMBA (65 mg DMBA/kg body mass) in sesame oil at 50–55 d of age. Mammary carcinomas ≥3 × 3 mm^2^ were counted at 15 wk post-treatment.

### Comparative genomics

Human orthologous regions and transcripts mapping to the delimited rat *Mcs3* and human orthologous loci were identified using the UCSC Genome Browser. The *R. norvegicus* reference genome sequence version RGSC 6.0/rn6 and *Homo sapiens* version GRCh38/hg38 were used. Breast cancer associated genes were identified by searching NCBI/PubMed using the respective *Mcs3*-nominated gene name and breast cancer as search terms. Databases at NHGRI-EBI (Welter *et al.* 2014) and GWAS Central (Beck *et al.* 2014) were searched for variants located in *Mcs3* orthologous regions that had *p* values of 1 × 10^−3^ or less for association to breast cancer.

### Statistical analysis

Mammary carcinoma multiplicity data were analyzed by comparing congenic strain phenotypes to a susceptible WF/NHsd phenotype. First, mammary carcinoma multiplicity phenotypes for all lines were compared using the Kruskal-Wallis nonparametric test to protect for multiple comparisons. Following a significant Kruskal-Wallis test, which was *p* < 0.0001, select group comparisons were made by two-tailed Mann-Whitney nonparametric tests corrected for ties; *p* values ≤ 0.05 were considered statistically significant.

### Data availability

Strain WF.Cop D is available upon request. Table S1 contains sequence and location information for primers to amplify microsatellite markers on *RNO1*. Table S2 contains sequence and location information for primers to amplify *RNO1* SNV containing sequences. File S1 contains a list of *Mcs3*-nominated breast cancer susceptibility candidate genes.

## Results

### Physical confirmation and positional mapping of Mcs3 With WF.Cop congenics

**Figure 1:**
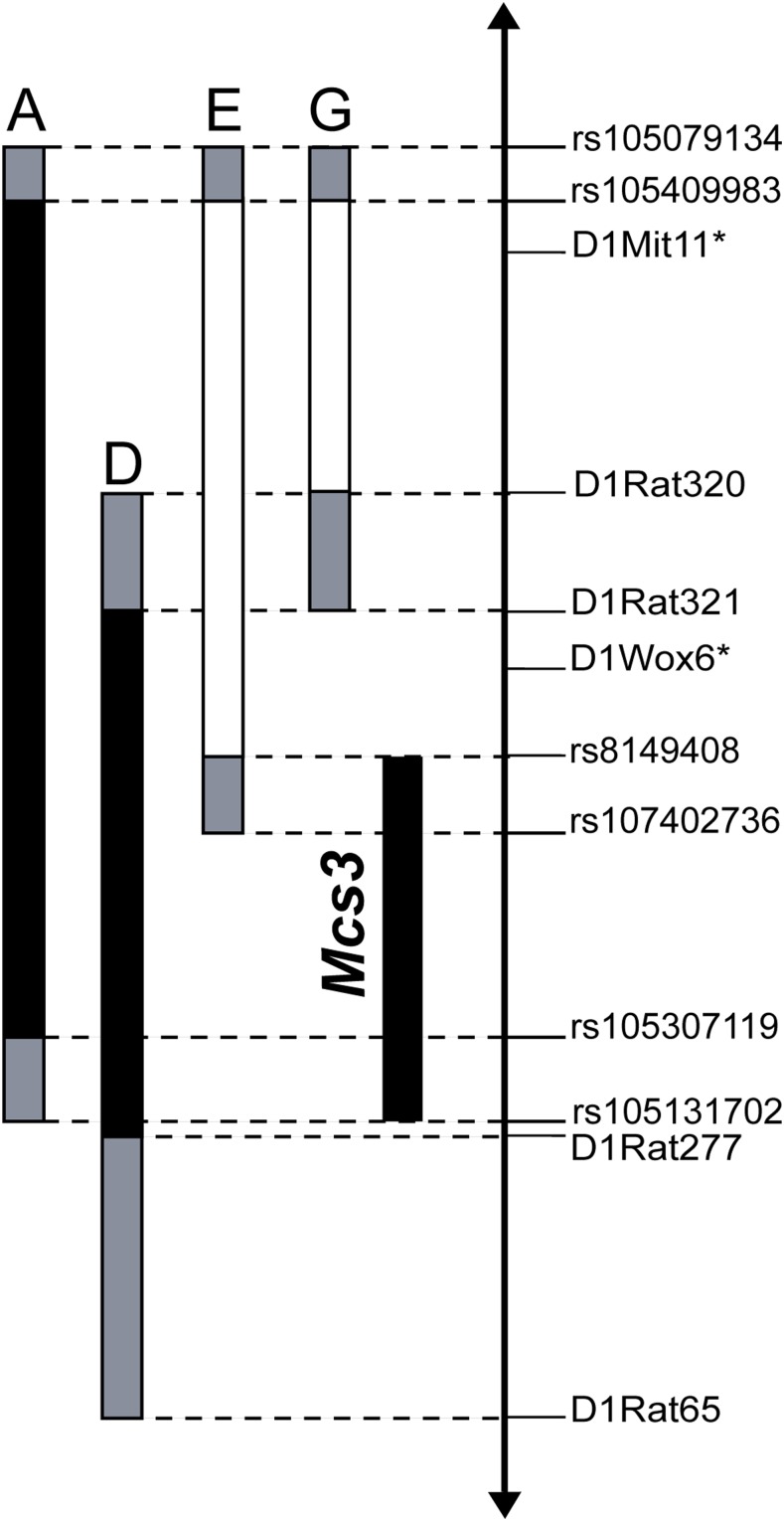
Map of WF.Cop congenic lines that delimit Rat *Mcs3* to 27.8 Mb of *RNO1*. WF.Cop congenic lines used to physically confirm and map *Mcs3* are represented by vertical bars that, respectively, define the Cop segment of *RNO1* introgressed into a WF/NHsd strain genetic background. Black bars represent Cop alleles in congenic lines with an *Mcs3*-associated mammary carcinoma resistant phenotype, whereas white bars represent congenic lines with a susceptible phenotype. Gray bars at the ends of congenic segments mark regions of unknown genotype. The vertical axis represents a segment of *RNO1* defined by relative locations of informative genetic markers shown as horizontal tick marks. Asterisks indicate the peak LOD score markers from the original QTL scan (Shepel *et al.* 1998).

The *Mcs3* QTL was predicted to exist on *RNO1* by the laboratory of M. N. Gould in a previous study (Shepel *et al.* 1998). Figure 1 is a map of WF.Cop congenic strains used in the present study to physically confirm and positionally map the location of *Mcs3*. Table 1 contains DMBA-induced mammary carcinoma susceptibility phenotypes (mean ± SD mammary carcinomas per rat) of the congenic lines depicted in Figure 1. Congenic lines A (*n* = 30) and D (*n* = 19) developed 3.4 ± 2.2 and 2.8 ± 2.3 mammary tumors per rat, respectively. Mammary cancer susceptible WF females (*n* = 12) developed 6.3 ± 3.8 DMBA-induced mammary carcinomas per rat. Females from lines A and D developed significantly less mammary tumors per rat compared to susceptible WF control females (*p*-values = 0.019 and 0.015, respectively). Reduced susceptibility lines A and D contained partially overlapping segments of Cop *RNO1*, and were not different from each other with respect to DMBA-induced tumor multiplicity (*p*-value = 0.198). This suggests that these congenic lines likely contain the same QTL. Thus, the reduced susceptibility phenotypes of both lines, A and D, physically confirm the *Mcs3* locus.

**Table 1:** Mammary carcinoma multiplicity phenotypes of WF.Cop *RNO1* congenic and WF/NHsd females

Strain	Genetic Markers Spanning Cop *RNO1* Maximal Segment	Phenotype Mean ± SD	*N*	*p*-Value*^a^*
A	*rs105079134* to *rs105131702*	3.4 ± 2.2	30	0.019
D	*D1Rat320* to *D1Rat65*	2.8 ± 2.3	19	0.015
E	*rs105079134* to *rs107402736*	6.6 ± 3.4	25	0.935
G	*rs105079134* to *D1Rat321*	8.1 ± 3.4	29	0.334
WF/NHsd	NA	6.3 ± 3.8	12	—

WF, Wistar Furth; Cop, Copenhagen; SD, standard deviation; NA, not applicable.

a *p*-values from Mann-Whitney *post hoc* tests comparing each congenic line phenotype to a susceptible WF/NHsd phenotype following a statistically significant Kruskal-Wallis test with *p*-value < 0.0001.

Congenic lines E (*n* = 25) and G (*n* = 29) developed 6.6 ± 3.4 and 8.1 ± 3.4 mammary tumors per rat, respectively. Neither line was significantly different in tumor multiplicity compared to susceptible WF rats (*p*-values = 0.935 and 0.334, respectively). Thus, neither line E nor line G contains an independently acting mammary carcinoma susceptibility QTL with an effect on the WF susceptibility phenotype.

The region of overlap between Cop *RNO1* segments that indicated a presence of at least one independently acting *Mcs3* allele (lines A and D) in combination with nonoverlap between these segments and the Cop *RNO1* segment contained in line E, which did not indicate the presence of an independently acting Cop allele, was used to delimit the *Mcs3* QTL interval (Figure 1). Congenic *Mcs3*-resistance-associated lines A and D overlapped each other minimally from markers *D1Rat321* to *rs105307119*, and maximally from *D1Rat320* to *rs105131702*. Susceptible line E minimally overlapped resistance-associated line A from *rs105409983* to *rs8149408* and resistance-associated line D from *D1Rat320* to *rs8149408*. Considering the phenotypes and relevant regions of overlap and nonoverlap of lines A, D, and E, the *Mcs3* QTL was delimited to a 27.8 Mb region of *RNO1* that spans from SNVs *rs8149408* to *rs105131702*. Respectively, these SNVs physically mapped to *RNO1* base positions 143,700,228 and 171,517,317 of the rat reference genome build RGSC 6.0/rn6.

Results of the original QTL scan predicted two LOD score peaks on *RNO1* at markers *D1Mit11* and *D1Wox6* (Shepel *et al.* 1998). These peak markers are denoted with asterisks in Figure 1. Congenic line E had the Cop genotype at both peak markers, but did not have an *Mcs3*-associated resistance phenotype, as would be predicted based on the original QTL scan. The phenotype of congenic line G, which was derived from line E and contained the Cop allele at *D1Mit11* and the WF allele at *D1Wox6*, was measured to determine if epistatic QTL might be responsible for the susceptibility phenotype of line E. The susceptibility phenotypes of lines E and G were not significantly different when compared to each other (*p*-value = 0.182). Thus, we did not uncover evidence of an epistatic interaction to potentially explain the susceptibility phenotype of line E.

### Human orthologous regions to the rat Mcs3 QTL

The delimited rat *Mcs3* QTL from *rs8149408* to *rs105131702* of *RNO1* was found to align to four syntenic regions of human chromosomes 11 and 15 and four other regions of the human genome that contained a single gene (Table 2). The UCSC genome browser (https://genome.ucsc.edu) was used to identify known genes at rat *Mcs3* and orthologous human loci (Kent *et al.* 2002; Karolchik *et al.* 2004). These genes are listed in File S1. There were 310 rat and 287 human genes annotated in the respective genomic intervals. At least 198 of these genes were in common or orthologous between these species.

**Table 2:** Minimum-number or names of genes at human loci containing rat *Mcs3* orthologous sequence

Human Locus	Position*^a^*	Gene Transcripts (Minimum Number or Name)		
		Human	Rat	Human/Rat Orthologs
*15q25.2*	15:83134545–84130720	6	5	5
*15q25.1*–*15q25.2*	15:80005820–82285404	22	13	13
*11q13.4*–*11q13.5*, *11q14.1*–*11q14.3*	11:71915764–89617253	142	103	87
*11p15.4*	11:3609839–7196087	113	186	90
*Xq28*	X:154886349–154888061	*F8A1*	*F8a1*	1
*10q23.33*	10:94762624–94853260	*CYP2C19*	*Cyp2c6v1*	1
*11p11.12*	11:49146635–49208670	*FOLH1*	*Folh1*	1
*2p16.3*	2:51696250–51787033	*LOC730100*	0	0
Total		287	310	198

Rat *Mcs3* from SNVs *rs8149408* to *rs105131702* or *RNO1*:143700228–171517317 (RGSC 6.0/rn6).

a Human genome reference GRCh38/hg38.

Public databases, namely the NHGRI-EBI catalog (https://www.ebi.ac.uk/gwas) (Welter *et al.* 2014), GWAS Central (www.gwascentral.org) (Beck *et al.* 2014), NCBI PubMed (https://www.ncbi.nlm.nih.gov/pubmed), and the Cancer Portal of Rat Genome Database (RGD) (https://rgd.mcw.edu), were searched to identify genes and genetic variation at *Mcs3* orthologous human genome regions that have been associated with breast carcinoma susceptibility and development. Table 3 contains a list of female breast cancer associated genes and amplified regions that have *Mcs3*-nominated rat orthologs. Rat *Mcs3* was found to contain sequence orthologous to human *11q13/14*, which contains multiple genes and is amplified in a subset of female breast carcinomas with poor prognosis (Tsuda *et al.* 1989). In addition to a functional correlation with breast cancer development, studies reporting genetic associations of *CYP2C19* variants with breast cancer susceptibility have been published (Justenhoven *et al.* 2009; Sangrajrang *et al.* 2009; Gan *et al.* 2011).

**Table 3:** Rat *Mcs3*-nominated human genes and amplified region correlated with breast cancer development

ID	Name	Locus	RefSeq on Function	Reference
Genes				
* ILK*	*Integrin-linked kinase*	*11p15.4*	Serine/threonine kinase that regulates integrin-mediated signaling	Yang *et al.* (2013)
* IL18BP*	*Interleukin 18 binding protein*	*11q13.4*	Inhibitor of proinflammatory cytokine IL18	Yang *et al.* (2015)
* EMSY*	*EMSY*, *BRCA2 interacting transcriptional repressor*	*11q13.5*	Repression of BRCA2-mediated DNA repair	Hughes-Davies *et al.* (2003), Madjd *et al.* (2014)
* PAK1*	*p21 (RAC1) activated kinase 1*	*11q13.5*–*q14.1*	Serine/threonine kinase that regulates cell motility and morphology	Adam *et al.* (2000), Wang *et al.* (2005), Bostner *et al.* (2007)
* RSF1*	*Remodeling and spacing factor 1*	*11q14.1*	Histone chaperone involved in RSF chromatin-remodeling complex	Ren *et al.* (2014)
* GAB2*	*GRB2 associated binding protein 2*	*11q14.1*	Adapter protein involved in signal transduction with a known role in PI3K activation	Bocanegra *et al.* (2010)
* CYP2C19*	*Cytochrome P450 family 2 subfamily C member 19*	*10q23.33*	Monooxygenase involved in xenobiotic and estrogen metabolism	Justenhoven *et al.* (2009), Sangrajrang *et al.* (2009), Gan *et al.* (2011)
Amplified region
* 11q13/14*			Amplified in a subset of breast cancers that are typically ER+ and have a poor prognosis	Tsuda *et al.* (1989)

Genome-wide significant associations (*p* values < 10^−7^) between human genetic variants located in *Mcs3*-orthologous multigene regions and female breast cancer risk have not been reported, but potentially associated variants with *p* values < 10^−3^ have been reported (Table 4).

**Table 4:** Rat *Mcs3*-nominated human variants potentially associated with breast cancer susceptibility

Variant	Locus	Base Position (hg38)	Genomic Context/Position	*p*-Value for Association	Reference
*rs1435808*	*2p16.3*	*chr2*:52,036,315	Intronic/*LOC730100*	0.000642	Hunter *et al.* (2007)
*rs6733295*		*chr2:52,040,189*	Intronic/*LOC730100*	0.000959	Hunter *et al.* (2007)
*rs4352262*		*chr2:53,374,593*	Intergenic/between *LOC730100* and *ASB3*	0.000893	Hunter *et al.* (2007)
*rs10168550*		*chr2:53,385,857*	Intergenic/between *LOC730100* and *ASB3*	0.000381	Hunter *et al.* (2007)
*rs7126870*	*11p15.4*	*chr11:3868829*	Intronic/*STIM1*	0.000898	Hunter *et al.* (2007)
*rs1459952*	*11q14.1*	*chr11:81871697*	Intergenic/between *LOC101928944* and *MIR4300HG*	0.000562	Kibriya *et al.* (2009)
*rs3793949*		*chr11:83847695*	Intronic/*DLG2*	0.000702	Hunter *et al.* (2007)
*rs11235127*	*11q14.2*	*chr11:87377502*	Intergenic/between *THEM135* and *LOC105369423*	0.00005	Easton *et al.* (2007)
*rs12269979*		*chr11:87960152*	Intergenic/between *LOC105369423* and *RAB38*	0.000439	Hunter *et al.* (2007)
*rs6495623*	*15q25.2*	*chr15:81848308*	Intergenic/between *TMC3* and *MEX3B*	0.000871	Hunter *et al.* (2007)

## Discussion

Rat *Mcs3* was physically confirmed and delimited to a 27.8 Mb segment of rat chromosome 1. Rat *Mcs3* is the last of the known Cop rat mammary carcinoma resistance-associated QTL (*Mcs1-3*) to be physically confirmed. In their linkage analysis predicting the *Mcs3* QTL, Gould’s group reported that *Mcs3* heterozygous females had, on average, a 42% reduction in mammary carcinoma multiplicity compared to the WF phenotype, and females homozygous for the *Mcs3* Cop allele had an 84% reduction in mammary tumor number (Shepel *et al.* 1998). Thus, they appropriately concluded that there was no dominance effect at the *Mcs3* QTL. While we did not test heterozygous females in our study, we observed that the *Mcs3* Cop allele reduced the mammary carcinoma susceptibility phenotype of the highly susceptible WF strain from 46 to 65% when homozygous. The discrepancy between homozygous genotypes in these two studies may be due to effects of Cop resistance alleles at other *Mcs* QTL present in the linkage analysis study, as the F_2_ females in that study were not required to be WF homozygous at *Mcs1* or *Mcs2* resistance-associated QTL. Another possibility is that *Mcs3* contains multiple independent QTL, and at least one of these was not contained in the congenic strains tested for our study.

The 27.8 Mb region of *RNO1* from SNVs *rs8149408* to *rs105131702* is the segment most likely to contain *Mcs3*; however, it is possible that *Mcs3* is more complex. For example, an interaction between one or more elements in the delimited region and elements outside of this region could be required to confer the *Mcs3*-associated phenotype. The delimited *Mcs3* segment is distal to both *D1Mit11* and *D1Wox6*, the markers with peak LOD scores from the original QTL scan, but within the predicted *Mcs3* QTL interval (Shepel *et al.* 1998). This is not the first time a rat *Mcs* locus has been mapped to a genomic segment that did not contain a peak marker of the original QTL scan. For example, neither *Mcs1b* nor *Mcs1c* QTL contain the peak LOD marker of the predicted *Mcs1* QTL (Haag *et al.* 2003; denDekker *et al.* 2012). The *Mcs3* QTL, as delimited here, overlaps the distal third of mouse *Mammary tumor susceptibility modifier 1* (*Mtsm1*) (Koch *et al.* 2007). Interestingly, the mouse *Mtsm1* segment overlapping rat *Mcs3* does not contain the peak LOD score marker defining the *Mtsm1* QTL. Furthermore, it is worth noting that *Mcs3* had one of the lowest LOD scores of *Mcs* QTL identified in linkage analyses using WF females as the susceptible strain (Shepel *et al.* 1998; Lan *et al.* 2001). Thus, interval mapping effectively predicted rat *Mcs* QTL, and, as expected, a wide genomic region of interrogation was required in congenic studies to pinpoint the location of each QTL for fine mapping.

Most importantly, this work provides information that is translatable to the genetic component of human breast cancer by using a versatile experimental organism that is highly relevant to female breast cancer. Different segments of the rat *Mcs3* locus align to four multigene human syntenic regions on chromosomes 11 and 15, and four single-gene loci at *Xq28*, *2p16.3*, *10p23.33*, and *11p11.12*. The rat *Mcs3* locus contains 310 annotated genes, and the orthologous human regions contain 287 annotated genes. To reduce the number of *Mcs3*-nominated candidate susceptibility genes, it will be necessary to fine-map the *Mcs3* locus. However, the human orthologous regions to rat *Mcs3* could be targeted for deep genetic analysis to determine if these regions contain risk-associated variants. In support of this direction, population-based genetic association studies have identified potentially associated variants at *Mcs3* orthologous loci *2p16.3*, *11p15.4*, *11q14.1*, *11q14.2*, and *15q25.2* (Easton *et al.* 2007; Hunter *et al.* 2007; Kibriya *et al.* 2009). This provides strong rationale for additional human studies to identify risk associated variants at these loci. Human genome targeted association studies require considerable resources, as a high density of variants and a large sample size are required to properly test each targeted locus. Guidance from both rat genomics and previous human genetic studies stands to increase the likelihood of finding positive associations. Thus, these human loci are excellent candidates for further genetic analyses to test a high density of variants for association to risk.

Discussion of the entire list of genes located within the *Mcs3* QTL, as currently defined, is not practical; however, it is notable that some *Mcs3*-nominated candidate susceptibility genes have known or suggested roles in breast cancer progression, diagnosis, or prognosis. This list includes *p21 (RAC1) activated kinase 1 (PAK1)*, *EMSY*, *BRCA2 interacting transcriptional repressor (EMSY)*, *remodeling and spacing factor 1 (RSF1)*, *GRB2 associated binding protein 2 (GAB2)*, *integrin-linked protein kinase (ILK)*, *interleukin-18-binding protein (IL18BP)*, and *cytochrome P450 family 2 subfamily C member 19 (CYP2C19)*.

The *EMSY*, *PAK1*, *RSF1*, and *GAB2* genes are located within the *11q13/14* breast cancer amplicon. Interestingly, the breast cancer oncogene *cyclin D1* (*CCND1*), which is also located within the *11q13/14* amplicon, is not contained within the rat *Mcs3* delimited region. The *EMSY* gene encodes a BRCA2 binding partner that silences a potential transcription activation domain of BRCA2, thereby repressing BRCA2-mediated DNA repair (Hughes-Davies *et al.* 2003; Moelans *et al.* 2010). Amplification of *EMSY* has been reported in 13% of breast cancers, and is associated with poor survival, especially in node negative breast cancer (Hughes-Davies *et al.* 2003). A recent study reported a significant positive association between EMSY expression and lymph node metastasis, as well as a larger tumor size (Madjd *et al.* 2014). The *PAK1* gene product has many roles in cancer, including breast cancer progression, development and maintenance of a metastatic phenotype of breast cancer cells, and a predictor of recurrence and tamoxifen resistance in postmenopausal breast cancer (Adam *et al.* 2000; Bostner *et al.* 2007; Kumar and Li 2016). Ectopic expression of activated Pak1 has been shown to induce mouse mammary tumors (Wang *et al.* 2005). High Rsf-1 expression is associated with breast cancer subtype and poor prognosis (Ren *et al.* 2014). Amplification of *GAB2*, independent of *CCND1*, has been observed in breast carcinoma samples (Daly *et al.* 2002; Bocanegra *et al.* 2010).

A direct interaction between PAK1 and ILK proteins has been identified. The activity of ILK is regulated by PAK1 phosphorylation (Acconcia *et al.* 2007). Higher transcript levels of *ILK* have been found in human breast cancer tissue compared to adjacent nondiseased breast tissue (Yang *et al.* 2013). In the same study, breast cancer patients with more intense immunostaining for ILK had lower 5-yr survival than patients with low ILK levels (Yang *et al.* 2013).

Interleukin-18-binding protein (*IL18BP*) regulates the activity of IL-18, which has been shown to be higher in breast carcinoma tissue compared to tissue from patients with benign breast disease (Srabovic *et al.* 2011). Another study suggests that IL-18 enhances breast cancer cell migration (Yang *et al.* 2015). Due to a potential role of IL-18 in cancer progression and metastasis, an IL18BP-Fc has been developed to antagonize the effects of IL-18 (Cao *et al.* 2008).

Because of an established role in estrogen metabolism, *CYP2C19* variants have been tested for association with breast cancer risk. There is evidence that the CYP2C19*3 variant may be associated with breast cancer risk in Chinese Han women (Gan *et al.* 2011). Neither the CYP2C19*17 variant, which results in a rapid metabolizer phenotype, nor the CYP2C19*2 variant were associated with breast cancer risk in population-based studies of women of European descent; however, CYP2C19*17 was associated with decreased risk in women using hormone replacement therapy for >10 yr (Justenhoven *et al.* 2009, 2012; MARIE-GENICA Consortium on Genetic Susceptibility for Menopausal Hormone Therapy Related Breast Cancer Risk 2010). In a study of cancer recurrence in ER+ postmenopausal breast cancer cases, it was concluded that patients with a CYP2C19*2 allele may benefit more from tamoxifen therapy (Beelen *et al.* 2013).

In summary, rat *Mcs3* is an independently acting QTL located in a 27.8 Mb region on *RNO1* between *rs8149408* and *rs105131702*. Additional congenic studies and other genomic approaches will be necessary to reduce the *Mcs3*-nominated candidate susceptibility gene list and determine mechanisms of *Mcs3*-associated reduced susceptibility to mammary carcinomas. While an overwhelming list of *Mcs3*-nominated breast cancer susceptibility candidate genes was identified, a manageable number of orthologous human syntenic regions were found to warrant deeper analysis of these loci in human population-based genetic association studies. The fact that some of these regions contain variants potentially associated with breast cancer risk further supports the need to ultrafine-map these loci to determine if true positive associations to susceptibility exist.

## Supplementary Material

Supplemental material is available online at www.g3journal.org/lookup/suppl/doi:10.1534/g3.117.039388/-/DC1.
