# Supplementary material for: Physical Confirmation and Comparative Genomics of the Rat Mammary carcinoma susceptibility 3 Quantitative Trait Locus
Source: G3 (Bethesda). 2017 Apr 5;7(6):1767–73. doi: 10.1534/g3.117.039388 (PMC5473756; doi:10.1534/g3.117.039388)
Supplement: Supplementary file 2 [file 1767TableS1.docx]

| **Table S1. Primers for Microsatellite Markers**^†^ **on *RNO1*** | | | |
| --- | --- | --- | --- |
| **Marker ID** | **Forward Primer Sequence** | **Reverse Primer Sequence** | **PCR Amplicon Position^*^** |
|  |  |  |  |
| *D1Rat27* | GGGCAAGCAAAGTACATGGT | TCTCTCCAGCTGCAGGATTT | 94201400- 94201552 |
| *D1Mit30* | TGTCTTGGCCTCTGATTTCA | TGCTGTGTGGACGGAGATAA | 99983293- 99983705 |
| *D1Rat214* | GATTTTCAATCTTTTAGCAAGCA | TTTCAGTGACAACCAGGCTG | 105499947- 105500046 |
| *D1Mit11* | ATAAGCCAGCCCCCATTC | CCTACTGAAAGTGAAATGTCTGG | 108057233- 108057505 |
| *D1Rat32* | TGAGCCATTGAGTTGTGAGC | TGTTTTCCAAATGAAGCGTG | 195598053-195598217 |
| *D1Rat381* | TCAAAAGGCTAAGGCAGGAA | AGACAAAAATGGAGGCCTCA | 121241973-121242176 |
| *D1Rat320* | CTCCACATGCACAGGCATAT | CTCCTAAAGGTCCCATTGCA | 125875758-125875986 |
| *D1Rat321* | TGCACCAAATTTTCTATTCCA | ACATTTGCCTTTTTGGACAT | 134120332-134120480 |
| *D1Rat36* | TTCCTGGGGTACTCCCAC | TTCCTCTCCTTCAACTCCTCC | 135022396-135022531 |
| *D1Wox6* | CCCCATCTATCTATCCAACGG | CTCTGGGATGCTTTGTGAAGG | 137787261-137787460 |
| *D1Rat382* | GGCCGAATGCTTTCAATAGA | GGCATACATGCTCAAACTGC | 137503081-137503201 |
| *D1Rat350* | CCAGATGAAGGCTGATGGAC | ACACGGTATTCAATCAGCCC | 141580935-141581089 |
| *D1Rat173* | GATCCCTTGACAAGCATGGT | GATGGAGGCAGTTTTTCCAA | 152519382-152519547 |
| *D1Mgh8* | CCTCTGGATTCTGCCAGAAG | TTTCAAATGTACAGGCTGAAACA | 163796316-163796432 |
| *D1Rat277* | TCTGGTCTTTACATGTATGTGCA | TTCACATCAGTTTTGGCCAC | 171716519-171716714 |
| *D1Rat243* | TCAACTCCCAGGGATTGTTC | CCCTGCTCTAACCAGCAGTC | 178985175-178985413 |
| *D1Rat65* | TGAAGGAGAGCCAGGAATTG | CAGTCTGGGGGTAAGCAAGA | 197209931-197210059 |
| *D1Rat290* | GGCTGAGTTTTTCTCTGACACTG | GGTCTCACTTCAGGGAGCAG | 207723700-207723901 |
| ^†^Microsatellite marker genotypes were determined using 3% high resolution agarose  ^*^Position is *Rattus norvegicus* *Chr1* genome build version 6.0 | | | |
|  | | | |
|  | | | |
|  | | | |
