# Supplementary material for: Physical Confirmation and Comparative Genomics of the Rat Mammary carcinoma susceptibility 3 Quantitative Trait Locus
Source: G3 (Bethesda). 2017 Apr 5;7(6):1767–73. doi: 10.1534/g3.117.039388 (PMC5473756; doi:10.1534/g3.117.039388)
Supplement: Supplementary file 3 [file 1767TableS2.docx]

| **Table S2. Primers to amplify *RNO1* SNV containing sequence** | | | | | | | | | |
| --- | --- | --- | --- | --- | --- | --- | --- | --- | --- |
| **dbSNP ID** | **Ensembl ID** | **Forward Primer** | **Reverse Primer** | **Amplicon Size** | **Amplicon Position*** | **Variant Position^*^** | **Variant** | **COP** | **WF** |
| *rs105079134* | *ENSRNOSNP2783780* | TTGTCCCTGAGCCTCG | GCCCGTCTCCACTTCT | 251 | 99464118-99464368 | 99464264 | C/T | CC | TT |
| *rs105409983* | *ENSRNOSNP2783800* | GGAGTCTGGGCACTGT | ACACCCAGCTAGCAGG | 278 | 104612966-104613243 | 104613093 | T/A | TT | AA |
| *rs8144718* | *ENSRNOSNP2784076* | GTGAGCAGCTGTTGGG | GGGAACTCACACCGGA | 276 | 142931545-142931820 | 142931722 | T/C | TT | CC |
| *rs8149408* | *ENSRNOSNP2784088* | AGTATCTGCCCGGTGG | GGCAAACCGTCCTGAAA | 155 | 143700164-143700318 | 143700228 | G/A | AA | GG |
| *rs107402736* | *ENSRNOSNP2784133* | TGCTTCGCCTTAACCTG | TGCATGTCAGAAGGGAGA | 330 | 150929449-150929778 | 150929594 | G/A | GG | AA |
| *rs105307119* | *ENSRNOSNP2784267* | TGACCTCCGTGCTACC | ACCTGGTGTCGCTTCA | 253 | 165593736-165593988 | 165593914 | T/A | AA | TT |
| *rs105131702* | *ENSRNOSNP2784324* | CTGTCACCCCAGCACT | TGATTTGTCCCGGGGA | 281 | 171517184-171517464 | 171517317 | T/C | TT | CC |
| ^*^Position is *Rattus norvegicus* *Chr1* genome build version 6.0 | | | | | | | | | |
